# Self-Referenced Optical Fiber Sensor for Hydrogen Peroxide Detection Based on LSPR of Metallic Nanoparticles in Layer-by-Layer Films

**DOI:** 10.3390/s19183872

**Published:** 2019-09-07

**Authors:** Javier Goicoechea, Pedro J. Rivero, Samuel Sada, Francisco J. Arregui

**Affiliations:** 1 Department of Electrical and Electronic Engineering, Nanostructured Optical Devices Laboratory, Public University of Navarre, Campus Arrosadía S/N, 31006 Pamplona, Spain; 2 Institute of Smart Cities (ISC), Public University of Navarre, Campus Arrosadía S/N, 31006 Pamplona, Spain; 3 Engineering Department, Public University of Navarre, Campus Arrosadía S/N, 31006 Pamplona, Spain; 4 Institute for Advanced Materials (INAMAT), Public University of Navarre, Campus Arrosadía S/N, 31006 Pamplona, Spain

**Keywords:** optical fiber sensor, Localized Surface Plasmon Resonance (LSPR), silver nanoparticles, gold nanoparticles, hydrogen peroxide detection, self-referenced sensor

## Abstract

Intensity-based optical fiber sensors are one of the most studied sensor approaches thanks to their simplicity and low cost. Nevertheless, their main issue is their lack of robustness since any light source fluctuation, or unexpected optical setup variation is directly transferred to the output signal, which, significantly reduces their reliability. In this work, a simple and robust hydrogen peroxide (H_2_O_2_) optical fiber sensor is proposed based on the Localized Surface Plasmon Resonance (LSPR) sensitivity of silver and gold metallic nanoparticles. The precise and robust detection of H_2_O_2_ concentrations in the ppm range is very interesting for the scientific community, as it is a pathological precursor in a wide variety of damage mechanisms where its presence can be used to diagnose important diseases such as Parkinson’s disease, diabetes, asthma, or even Alzheimer’s disease). In this work, the sensing principle is based the oxidation of the silver nanoparticles due the action of the hydrogen peroxide, and consequently the reduction of the efficiency of the plasmonic coupling. At the same time, gold nanoparticles show a high chemical stability, and therefore provide a stable LSPR absorption band. This provides a stable real-time reference that can be extracted from the spectral response of the optical fiber sensor, giving a reliable reading of the hydrogen peroxide concentration.

## 1. Introduction

The use of optical fiber sensors has attractive and significant advantages such as being light-weight, a small size, biocompatibility, a remote sensing capability, immunity to electromagnetic interferences, and the possibility of multiplexing several signals [1]. Due to this, optical fiber sensors have been studied in biomedical, clinical, environmental protection, healthcare and pharmaceutical research areas, among others [2]. In this sense, the development of precise and reliable optical fiber sensors for the analytical determination of hydrogen peroxide (H_2_O_2_) is of great interest in the biosensing field because hydrogen peroxide is a highly reactive oxidant which plays an important role in many biological, chemical, environmental, or even industrial processes. A representative example is that H_2_O_2_ is considered as a toxic by-product of many biological oxidases, being a very clear indicator of important diseases such as Parkinson’s disease, Alzheimer’s disease, asthma, diabetes, atherosclerosis, and breast cancer, among others [3,4]. Several works can be found in the bibliography related to the optical fiber hydrogen peroxide detection by using sensitive coatings on the tip of a multimode optical fiber [5,6] or onto bent optical fibers [7]. Those approaches typically take the optical reference at the beginning of the experiment, and the measurements are based on the light intensity variation with respect to this initial reference. Consequently, the stability of the measurement conditions is a critical issue in most of the intensity-based optical fiber sensor approaches.

However, the possibility of controlling the composition and structure of the sensitive coatings at the nanoscale level opens the door to the modification of their optical properties thanks to new nanoscale-based phenomena, making an improvement possible in the sensor’s response time and sensitivity [8]. In regards to this, some nano-structured approaches can be found in the bibliography, for example, an optical fiber core coated with only an Ag-film [9] or an Ag-film combined with Ag nanoparticles (AgNP) embedded in polyvinyl alcohol (PVA) [10] for hydrogen peroxide detection. 

Among other possibilities, one of the most interesting deposition techniques for the fabrication of nanostructured sensitive coatings is the Layer-by-Layer (LbL) nano-assembly. Using this technique, it is possible to precisely adjust the final thickness of the nanocoatings by adjusting the deposition parameters (concentration of the polyelectrolytes, pH, ionic strength of the dipping solutions, or immersion time, among other parameters) [11,12] and, at the same time, controlling the composition and structure of the multilayer film [13,14,15]. According to this, several optical fiber sensor approaches based on the implementation of the LbL technique for the fabrication of sensitive nanocoatings have been presented for hydrogen peroxide detection [16,17,18,19]. In some works, the sensors were based on the color change of a redox dye, such as by immobilizing Prussian Blue within a LbL multilayer, and the sensing mechanism relies on the change in the intensity of the reflected light when Prussian Blue is oxidized back to a blue state due to the presence of hydrogen peroxide. 

The use of metal nanoparticles for sensing applications is of great interest due to their attractive optical properties which is associated to their Localized Surface Plasmon Resonances (LSPR). The extremely confined electromagnetic fields induced by the LSPR phenomenon provide a highly sensitive tool to detect small changes in the resultant dielectric environment around the nanoparticles. The LSPR of a specific metallic nanoparticle has also a specific optical attenuation band, a distinctive spectral response which experiences a spectral shift depending on these small changes in the surrounding environment of the metallic nanoparticle and constitutes an important platform for sensing or biosensing applications [20]. Moreover, since the sensing mechanism of approaches relies on changes in the optical spectral response, they are also more robust than the previously mentioned works based on intensity measurement. However, these devices also suffer undesired fluctuations because the LSPR band can shift due to high sensitivity to the surrounding media around the nanoparticles. 

In this work, we propose to use the LbL nano-assembly as a tool that allows the immobilization of different types of metallic nanoparticles such as AgNPs and AuNPs into the same multilayer polyelectrolyte structure. This is a key technological improvement compared to the previous research work reported by our group [21], where an optical colorimetric sensor based on the UV-VIS analysis of colloidal dispersions of both types of metallic nanoparticles for hydrogen peroxide detection was presented. The use of this nanofabrication technique for the immobilization of the metallic nanoparticles makes good control over the distribution between neighboring metal nanoparticles possible, thereby enabling good control over the interparticle separation when the thickness is gradually increased during the fabrication process [22,23,24,25]. Those optical fiber sensors enable in situ measurements of the target analyte in real samples without any solution processing, which was necessary in previous works [21], thereby reducing the experimental errors that could lead to false measurements. Furthermore, since it is not necessary to dilute the sample, the measurement provided by the optical fiber sensor, will be directly from the H_2_O_2_ reading of the sample, thereby improving the effective LOD. Finally, such optical fiber sensor probes show the classical advantages of the optical fiber sensors, such as sensor portability, flexibility and small size, remote measurement and the future possibility of implementing this technology into LOC (Lab on a Chip) approaches combined with microfluidics. Each type of nanoparticle is associated to a different LSPR absorption band, in this case around 435 nm and 535 nm for AgNP and AuNPs, respectively. In addition, AgNPs and AuNPs show a remarkable difference in H_2_O_2_ sensitivity. Therefore, the idea is to detect the two different LSPR bands that experience different optical responses with an optical fiber sensor under the presence of hydrogen peroxide, as that combination can provide a stable real-time optical reference integrated in the optical response of the sensor. The monitoring of both LSPR could allow for cancelling the undesired fluctuations as a product of variations in the surrounding media that are not due to hydrogen peroxide. In other words, in this work a simple and low cost optical fiber sensor is presented that shows a remarkable robustness against light source fluctuations and any other unexpected issues that affect the intensity of the signal. To the best of our knowledge, this is the first time that a self-referenced optical fiber sensor based on a dual LSPR absorption band inherent to AgNPs and AuNPs is presented in the bibliography for hydrogen peroxide detection by using the Layer-by-Layer nano-assembly technique.

The organization of this paper is as follows: after a first NP-loaded LbL films characterization (Section 3.1), the performance of three different optical fiber LbL coatings are studied and compared. The first coating is a AgNPs only optical fiber sensor (Section 3.2), the second is a AuNPs LbL coating (Section 3.3), and the final one is a combined AgNPs and AuNPs LbL coating (Section 3.4). 

## 2. Experimental Procedure

### 2.1. Reagents and Materials

In this work, a cationic polyelectrolyte such as Poly(allylamine hydrochloride) (PAH, M_w_ = 56,000 g/mol) and an anionic polyelectrolyte such as Poly(acrylic acid sodium salt) 35 wt.% solution in water (PAA, M_w_ = 15,000 g/mol) have been selected for the fabrication of thin-films by using the Layer-by-Layer technique. Silver nitrate (AgNO_3_) and Gold (III) chloride trihydrate (HAuCl_4_. 3H_2_O) have been used as sources of the metallic ions, whereas a reducing agent such as dimethylaminoborane complex (DMAB) has been selected for the chemical reduction of the silver and gold ions into silver and gold nanoparticles, respectively. Hydrogen peroxide (H_2_O_2_) aqueous solutions used in this work were prepared by diluting a 30% H_2_O_2_ stock solution with de-ionized water. In order to determine the selectivity of the optical fiber sensor, the sensitive region has been exposed to ultrapure water and Hanks’ Balanced Salt Solution (HBSS), which was modified with calcium and magnesium, showed a pH range of 7.0–7.4, and was suitable for cell culture. In addition, the sensitive region has been also exposed to specific interfering agents (glucose or ascorbic acid) which can interfere in the corresponding measurement of the sensing analyte and produce false positive measurements [26]. The glucose solution was prepared at a concentration of 100 mM, and the ascorbic acid solution was prepared at a concentration of 400 ppm, respectively.

All the chemicals used in this work have been purchased from Sigma Aldrich and used as received without any further purification. Finally, plastic-clad silica fibers of 200/225 μm core/cladding diameter (FT200EMT) have been provided for Thorlabs Inc (Newton, NJ, USA). 

### 2.2. Synthesis of the Metallic Nanoparticles

A wet synthesis route based on the chemical reduction of both metallic salts (AgNO_3_ and HAuCl_4_. 3H_2_O) has been selected for the synthesis of AgNPs and AuNPs, respectively. In both cases, a water soluble polyelectrolyte such as PAA has been used as an efficient protective agent of the synthesized nanoparticles, showing good stabilization along their polymeric chains with good control over their corresponding morphologies (shape and size) [27,28]. An important advantage of this chemical synthetic route is that it is performed at room conditions and the resultant metallic nanoparticles (AgNPs, AuNPs) present a long-term stability without showing any sign of aggregation after long-storage.

For the synthesis of PAA capped AgNPs, denoted as PAA-AgNPs, 100 mL of 10 mM of PAA has been mixed with 0.100 mL of 0.1 N of AgNO_3_ with stirring for 1 h. After that, the addition of freshly 0.500 mL of 0.1 M DMAB to the previous solution was performed with constant stirring for 1 h. Then, in order to corroborate the synthesis of the capped-silver nanoparticles (PAA-AgNPs), a color change from transparent to yellow-orange was observed [29]. 

For the synthesis of PAA capped AuNPs, denoted as PAA-AuNPs, 100 mL of 10 mM of PAA has been mixed with 0.100 mL of 0.1 N of HAuCl_4_·3H_2_O and stirred for 1 h. Then the addition of freshly 0.025 mL of 0.1 M DMAB to the solution was performed with constant stirring for 1 h. A color change from pale yellow to red violet was observed, indicating that capped-gold nanoparticles (PAA-AuNPs) have been successfully synthesized [30]. 

### 2.3. Fabrication of the Layer-by-Layer Films

First of all, all the polyelectrolyte solutions of PAH, PAA-AgNPs and PAA-AuNPs were adjusted to pH 7.0 by the addition of a few drops of NaOH or HCl, respectively. Then, the Layer-by-Layer (LbL) films were performed by sequentially exposing the reference substrate (glass slides or optical fiber) to the cationic polyelectrolyte (PAH) and to the anionic polyelectrolyte capping the synthesized nanoparticles (PAA-AgNPs and PAA-AuNPs). The combination of a positive monolayer and a negative monolayer is called a bilayer henceforward. 

In this work, three different types of LbL coatings have been fabricated. Firstly, the LbL coating based on the immobilization of silver nanoparticles is presented which consists of multiple bilayers of [PAH/PAA-AgNPs]_n_, also noted as (AgLbL)_n_. Secondly, the LbL coating based on the immobilization of gold nanoparticles (AuNPs) is fabricated which consists of multiple bilayers of [PAH/PAA-AuNPs]_n_, also noted as (AuLbL)_n_. And finally, the LbL coating based on the immobilization of LbL stacks of both AgNPs and AuNPs is obtained which is composed of an accumulation of bilayers of [PAH/PAA-AgNPs]_n_ and [PAH/PAA-AuNPs]_m_. The resultant LbL coatings onto the optical fiber core are denoted as the sensitive region and all the LbL coatings have been fabricated by using a 3-axis Cartesian robot from Nadetech Innovations S.L. The LbL coatings show a good long-term stability and no oxidation have been observed at room conditions. Different numbers of bilayers have been used for each coating, and these numbers will be detailed in their correspondent sections. The optical absorbance of the LbL films onto glass substrates was characterized using a JASCO V-630 spectrometer. Finally, Scanning Electron Microscopy (SEM) images have been obtained by means of a HITACHI S4800 Field Emission SEM, running at 1 kV of acceleration voltage. The samples had been previously coated with an approximately 10 nm platinum layer in order to improve the conductivity of the sample, and hence the corresponding resolution. 

### 2.4. Optical Fiber Setup

**Figure 1:**
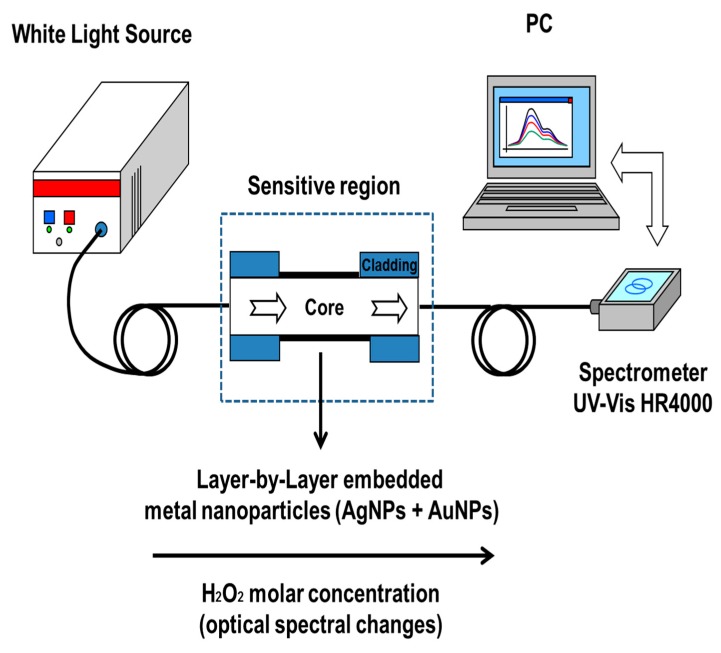
Schematic representation of the experimental setup for hydrogen peroxide detection as a function of the optical spectral changes associated to the metallic nanoparticles incorporated in the Layer-by-Layer coatings.

Optical absorbance spectroscopy has been used to monitor the LbL thin-film growth over the optical fiber as well as for the detection of the sensing analyte (H_2_O_2_) when the sensitive region is exposed to variable H_2_O_2_ concentrations. Although it is possible to get greater optical responses (higher sensitivities) with longer analyte exposure times, in this work this time was set to 10 min as the convention for all the sensors in order to get a fast characterization of the devices. A scheme of the setup used for the fabrication of the LbL coatings as well as for the detection of the sensing analyte is shown in Figure 1, using a halogen light source and an Ocean Optics USB2000 CCD (Largo, FL, USA) spectrometer. More details about optical fiber characterization can be found in references [31,32]. The optical response from all sensors was monitored during the LbL coating build-up and during the immersion in the samples. In this work, a 10 min sample immersion time has been used as a standard for all the sensor tests in order to get fast measurements with enough sensitivity, without compromising the stability of the LbL sensitive coatings.

## 3. Results and Discussion

### 3.1. Layer-by-Layer Films Loaded with NPs

**Figure 2:**
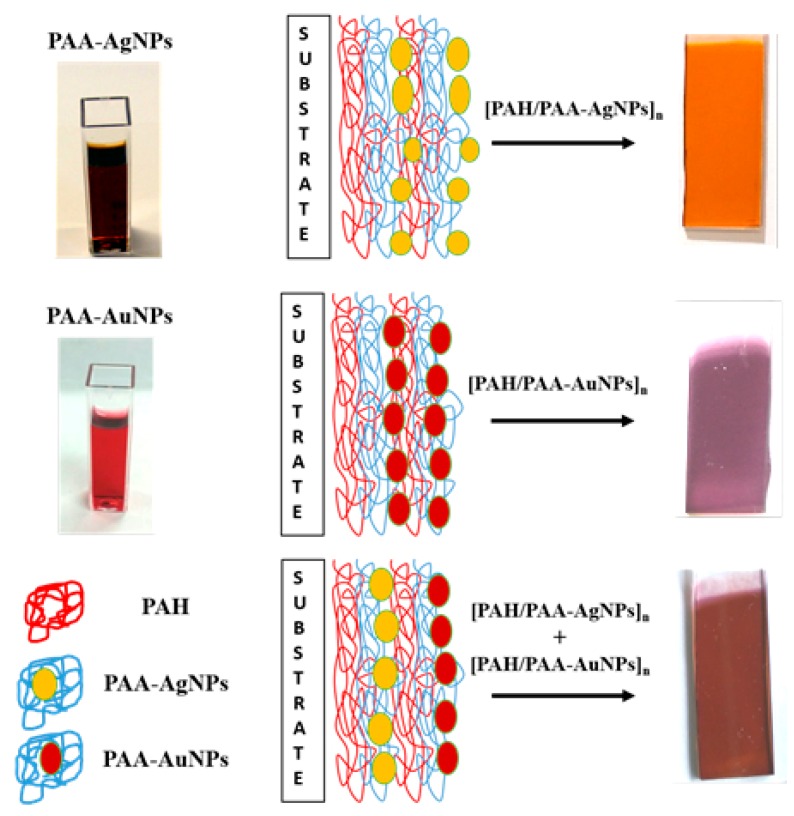
Aspect of the synthesized metallic nanoparticles (PAA-AgNPs, PAA-AuNPs), a schematic summary of the methodology for obtaining the LbL coatings based on the immobilization of metallic nanoparticles and final aspect of the LbL coatings on reference glass slides with a characteristic coloration after deposition process.

In an initial step, in order to corroborate an adequate embedding of the synthesized metallic nanoparticles into the thin films, glass slides have been firstly coated by using the Layer-by-Layer technique. It is important highlight that the goal is to have combined AgNPs and AuNPs LSPR bands in s single optical fiber sensor, nevertheless this previous characterization stage using glass substrates is necessary to study the properties LbL coatings, since many of the analytical techniques preformed here cannot be done using the optical fiber substrates. In Figure 2, an aspect of the synthesized PAA-AgNPs and PAA-AuNPs is presented in the form of a schematic representation of the fabrication process using the LbL technique on the reference substrate and the final aspect of LbL coatings composed of [PAH/PAA-AgNPs] (orange coloration), [PAH/PAA-AuNPs] (purple coloration) and [PAH/PAA-AgNPs] + [PAH/PAA-AuNPs] (red-wine coloration), respectively. 

**Figure 3:**
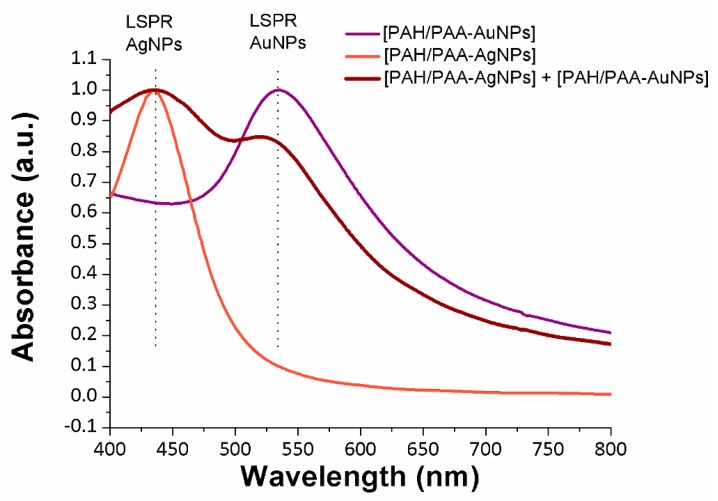
UV-Vis spectra changes of the LbL films based on the immobilization of metallic AgNPs (orange coloration), AuNPs (violet coloration) and mixture of both AgNPs and AuNPs (red-wine coloration), respectively.

The presence of the metallic nanoparticles into the LbL thin-films is visible to the naked eye. The samples show a similar coloration to the synthetized dispersions of AgNPs (orange) and AuNPs (purple). Afterwards, the LbL coated glass slides have been also characterized using UV-VIS in order to determine the location of the LSPR bands inherent to the metallic nanoparticles, as can be seen in Figure 3. 

A specific wavelength location of the LSPR bands displays a high dependence on the resultant shape and size of the metal nanoparticles [33]. In this sense, an absorption band centered at 435 nm can be clearly seen for the PAH/PAA-AgNPs coating which corresponds to the LSPR band of the AgNPs, whereas an absorption band centered at 535 nm is seen for PAH/PAA-AuNPs coating which corresponds to the LSPR band of the AuNPs. In addition, a dual band is observed for the PAH/PAA-AgNPs + PAH/PAA-AuNPs coating which corresponds to the LSPR bands of both types of metallic nanoparticles. The location of the LSPR bands of both AgNPs and AuNPs at these wavelength positions combined with the resultant coloration of the metallic nanoparticles clearly indicates the synthesis of metal nanoparticles with a specific morphology (mostly spherical shape and nanometric size) [34]. 

**Figure 4:**
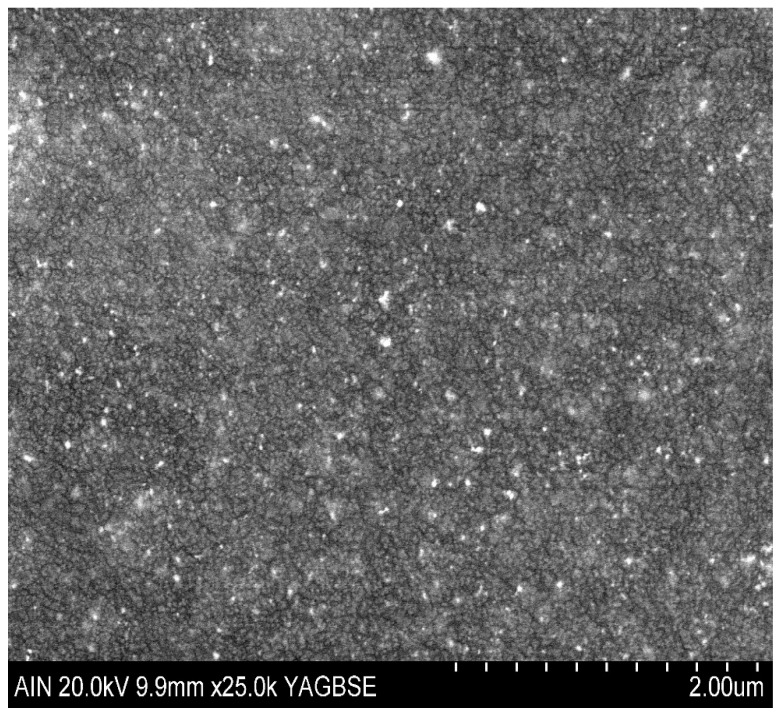
Backscattered electron image of the resultant surface morphology of the LbL coating composed of both types of nanoparticles (AgNPs and AuNPs, respectively). The light spots correspond to the metallic NPs.

Finally, in order to corroborate the distribution of the nanoparticles in the Layer-by-Layer films, the surface and coating morphology has been analyzed by using a Field Emission Scanning Electron Microscope (FE-SEM), as can be seen in Figure 4. A backscattered electron image has been employed for a better examination to determine the location of the resultant distribution of the metallic nanoparticles into the polymeric chains of the polyelectrolytes. A random and homogenous distribution of the synthesized metallic nanoparticles can clearly be observed inside the polymeric Layer-by-Layer coatings because the light spots observed in the Scanning Electron Microscope (SEM) image correspond to the synthesized metallic nanoparticles. 

### 3.2. AgNPs-Only Optical Fiber Sensor

**Figure 5:**
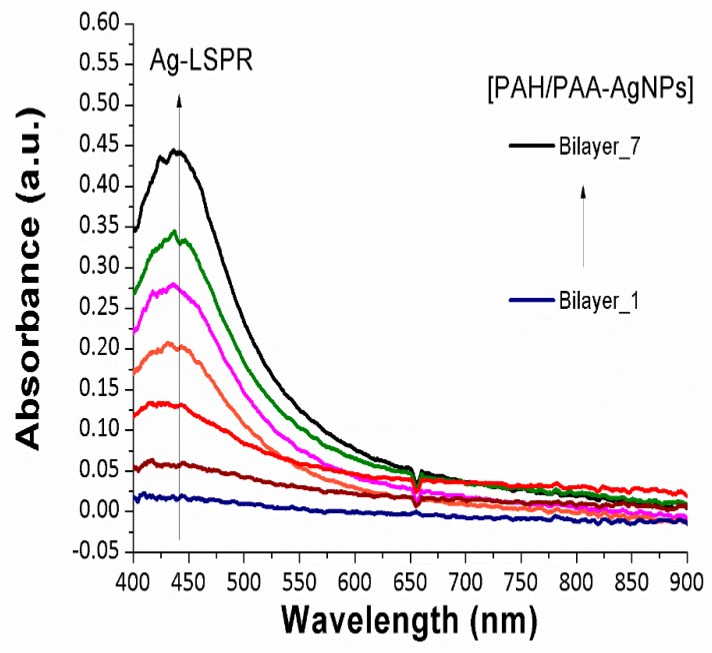
UV-Vis spectra during the fabrication of the Layer-by-Layer films based on the immobilization of metallic AgNPs. The curves are plotted from 1 up to 7 bilayers, respectively.

In the initial step, an optical fiber sensor based on the immobilization of only metallic silver nanoparticles (AgNPs) was fabricated. In Figure 5, the buildup onto the optical fiber claddingless sensitive region is presented for a total of 7 bilayers, denoted as [PAH/PAA-AgNPs]_7_. An absorption band at 435 nm can be observed which is associated to the LSPR phenomenon involving the AgNPs. The resultant intensity of this LSPR absorption band is gradually higher when the thickness coating onto the sensitive region is increased because a high amount of AgNPs are successively embedded into the LbL films during the fabrication process. 

**Figure 6:**
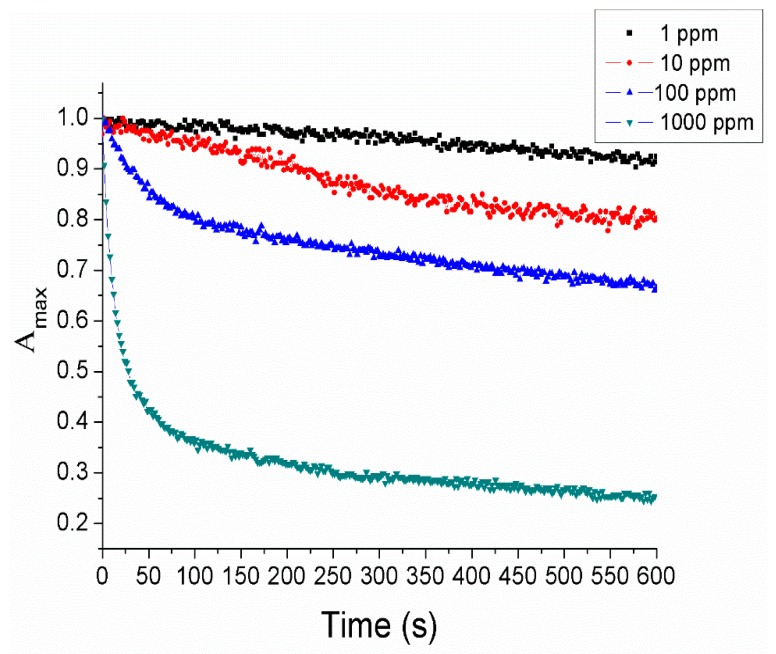
Evolution of the normalized maximum absorbance at 435 nm related to the Localized Surface Plasmon Resonance (LSPR)absorption band of the AgNPs when the sensitive region is immersed in different hydrogen peroxide concentrations of 1 ppm (black), 10 ppm (red), 100 ppm (blue), and 1000 ppm (green), respectively.

Once it has been confirmed that AgNPs have been deposited onto the optical fiber core, the next step has been to analyze the corresponding sensitivity of the device associated to the variation of the LSPR absorption band when the sensitive region is immersed in four different hydrogen peroxide concentrations (1000 ppm, 100 ppm, 10 ppm and 1 ppm, respectively). The results are summarized in Figure 6 where a variation of the normalized maximum absorbance (${\hat{A}}_{max}$) at 435 nm related to the LSPR absorption band is monitored up to a fixed period of time (600 s) for all analyte concentrations. In this figure, it can be clearly observed that the absorbance strength of the LSPR absorption band shows a high reduction in its maximum intensity when the analyte concentration is increased, which is more significant for the highest analyte concentration of 1000 ppm. This effect is attributed to the catalytic decomposition of hydrogen peroxide which induces a degradation of the PAA-AgNPs with the corresponding oxidation of silver nanoparticles to silver ions (Ag^+^), and as a result, the intensity of the LSPR absorption band is drastically decreased [21,35,36,37,38,39]. It is also shown in Figure 6 that the sensor dynamic (AgNPs oxidation rate) depends directly on the H_2_O_2_ concentration, which is consistent with the typical oxidation reaction dynamics. As the sensing principle of this approach is based in the AgNPs oxidation, in this work this reaction is considered to be irreversible and these sensors are not reusable. The possible regeneration of the AgNP LSPR band will be addressed in future research works. Another interesting aspect is that after immersion of the sensitive region for this highest concentration, no significant wavelength-shift was observed in the LSPR band, suggesting that there is no change in the aggregation state of the AgNPs and therefore meaning the polyelectrolytes of the LbL coating are perfectly encapsulating the resultant nanoparticles.

### 3.3. AuNPs-Only Reference LbL Coating

**Figure 7:**
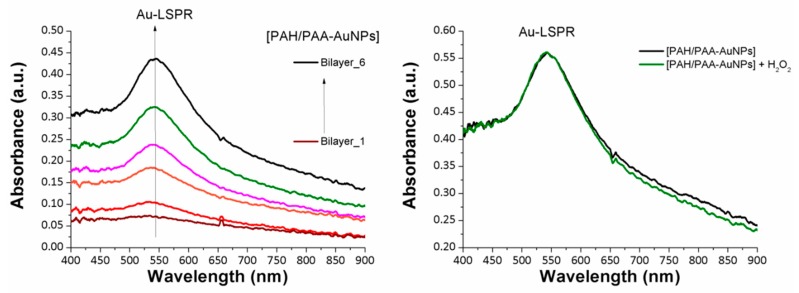
(**Left**) UV-Vis spectra of the optical fiber sensor as a function of the number of bilayers added during the fabrication of the Layer-by-Layer films. The curves are plotted from 1 up to 6 bilayers, respectively. (**Right**) UV-Vis spectra of the optical fiber sensor before (black line) and after (green line) exposure to a hydrogen peroxide concentration of 5000 ppm.

An optical fiber device based on the immobilization of only metallic gold nanoparticles (AuNPs) has been fabricated. For this coating, instead of seven bilayers, the number of bilayers has been adjusted to six, in order to have an equivalent intensity of the LSPR band compared to the previous AgLbL coating. In Figure 7 (left), the buildup of the Layer-by-Layer films is shown for the six bilayers and the LSPR inherent to the AuNPs centered at 535 nm can also been seen, which gradually increased with the resultant thickness coating. In addition, in order to corroborate the great stability of the AuNPs with the LbL films, the sensitive region has been immersed in a very high concentration of hydrogen peroxide (5000 ppm) for the same period of time (600 s), as can be seen in Figure 7 (right). The experimental results indicate that the intensity and the wavelength of the LSPR absorption band is unaltered for this high analyte concentration in comparison with the reference LbL coating, thereby showing very high stability compared with an optical fiber device composed of only AgNPs. This result is associated to the higher chemical stability of the AuNPs that remain unaltered after the addition of the sensing analyte. Consequently no changes in the Au-LSPR absorption band at 535 nm have been observed for this concentration, whereas for the LbL coating composed of only AgNPs showed a drastic reduction of the LSPR band because the AgNPs were gradually oxidized by the hydrogen peroxide. 

### 3.4. AgNPs Optical Fiber Sensor with AuNPs Reference

**Figure 8:**
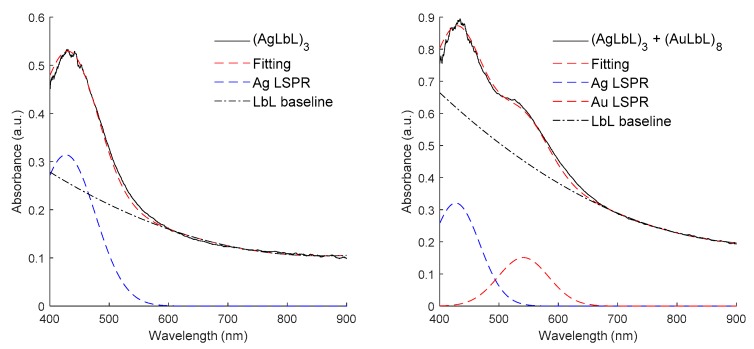
Sequential LbL adsorption of the sensitive coating onto the optical fiber core. (**Left**) Absorbance of (PAH/PAA+AgNPs)_3_ coating. The fitting of the Ag LSPR band is displayed using a Gaussian profile. (**Right**) Absorbance of (PAH/PAA-AgNPs)_3_+(PAH/PAA-AuNPs)_8_ coating. The contribution of the LSPR of both nanoparticle stacks is clearly visible.

In order to create a more robust optical fiber sensor, a sensor based on the immobilization of both types of metallic nanoparticles was fabricated. In this section, the number of bilayers for both Ag and Au LbL stacks has been experimentally adjusted in order to get a visible combination of both LSPR peaks in the combined coating. LbL stacks with less bilayers gave less sensitive optical fiber sensors, and LbL stacks with more bilayers showed an excess of the optical losses of the coatings, making the monitorization of both absorption LSPR bands more difficult. In Figure 7, the growth of the LbL films is shown after an initial addition of three bilayers of only AgNPs (left), noted as (AgLbL)_3_, and then the final buildup occurred after the addition of a second stack of eight bilayers of AuNPs (right) onto the previous AgNPs bilayers, denoted as (AgLbL)_3_+(AuLbL)_8_, respectively. The experimental optical response showed clear LSPR bands that were fitted by using a Gaussian profiles combined with the subtraction of LbL baseline related to the polyelectrolytes contribution (PAH, PAA) (processed using Matlab^®^). Figure 8 shows the good correspondence between the experimental data and the fitting model, making it possible to appreciate the isolated contribution of the LSPR absorption bands for AgNPs centered at 427 nm (dashed blue) and AuNPs centered at 541nm (dashed red), respectively. 

**Figure 9:**
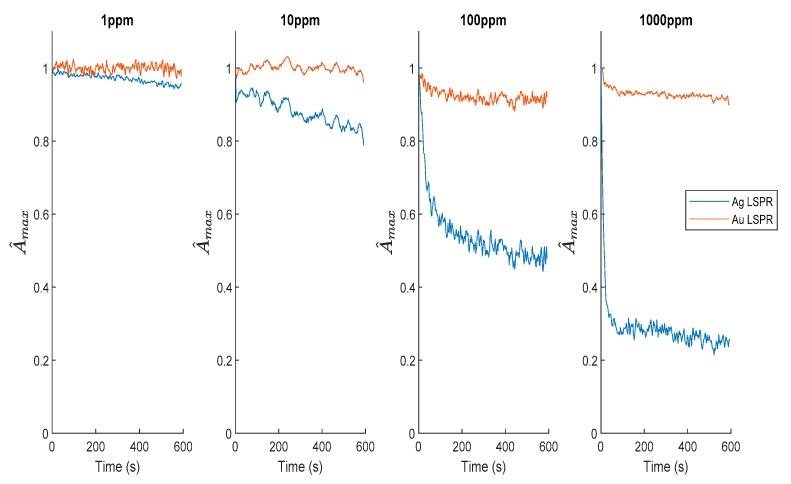
Sequential exposition of the optical fiber sensors to specific H_2_O_2_ concentrations of 1 ppm, 10 ppm, 100 ppm, and 1000 ppm, showing the variation of the LSPR band of AgNPs (blue plot) and the LSPR band of AuNPs (red plot), respectively.

Once the contribution of the both LSPR bands in the LbL films was confirmed, the next step was to analyze their corresponding reactivity to H_2_O_2_ (10, 100 and 1000 ppm), as can be seen in Figure 9. In this figure, the variation of the maximum absorbance related to the LSPR absorption bands as a function of the exposure time (600 s) are represented for both LSPR bands, the blue one corresponds to the AgNPs LSPR maximum and the red one corresponds to the AuNPs LSPR. The LSPR band associated to AuNPs was practically maintained at the same maximum absorbance for all H_2_O_2_ concentrations, whereas the maximum absorbance of the AgNPs was decreased by a high magnitude when the analyte concentration was increased. These experimental results show that the AuNPs LSPR signal can be used as a stable reference in order to get a differential measurement estimator that is noticeably more robust than the simple typical intensity-based measurements. In addition, the Ag LSPR maximum decreasing rate is clearly dependent on the peroxide concentration, in fact for 100 and 1000 ppm the response time is less than 1 min, while for the lowest concentration (1 ppm and 10 ppm) it is more than 10 min. As was previously discussed in Section 3.2, Figure 9 shows that the sensor dynamic (AgNPs oxidation rate) depends directly on the H_2_O_2_ concentration, which is consistent with the typical oxidation reaction dynamics. It seems that the degradation of the silver nanoparticles is slower and it did not reach its maximum during the 10 min of exposure time. This fact suggest that the sensitivity of these optical devices can be improved simply by extending their immersion time.

**Figure 10:**
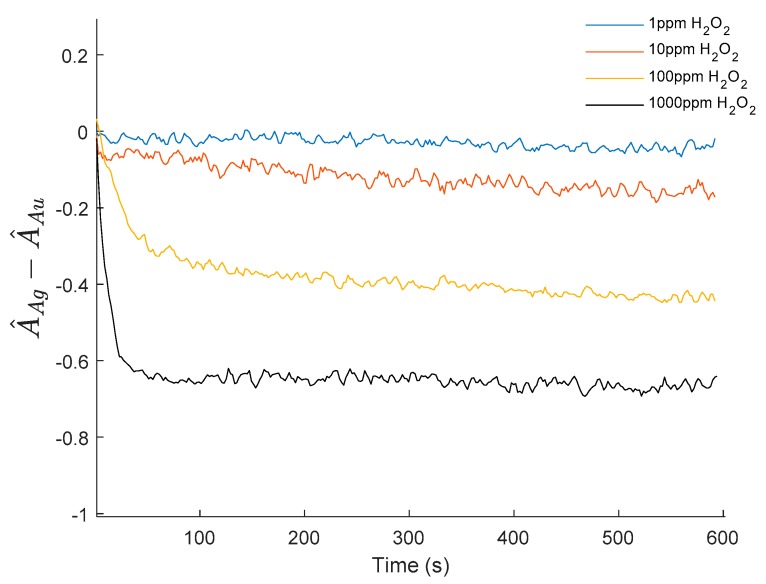
Time response of the difference between the normalized LSPR absorption bands of AgNPs and AuNPs. The response of this indicator is very similar to the direct read of the AgNPs LSPR maxima shown in Figure 5 for a simpler sensitive coating based only on AgNPs.

**Figure 11:**
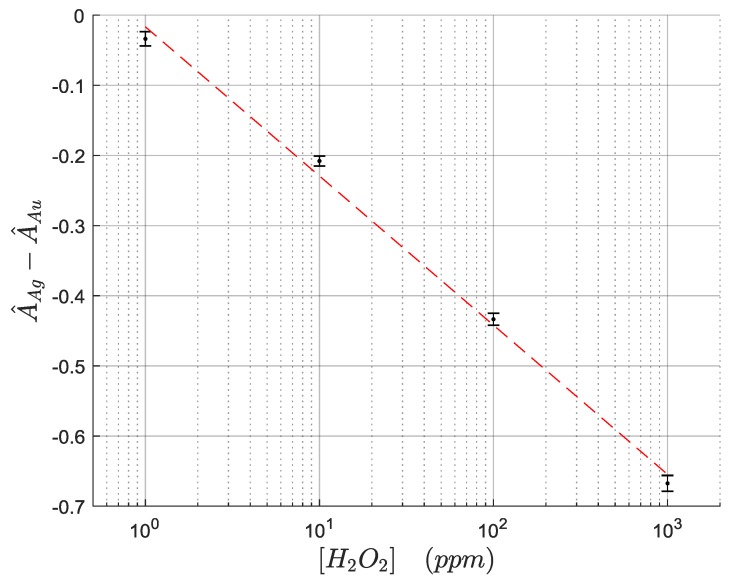
Calibration curve of the optical fiber sensors. The experimental measurements fit well with a linear relationship in a semilog plot, suggesting a logarithmic relationship of the measurement with the H_2_O_2_ concentration.

In this work the proposed measuring variable is the difference between the normalized peaks of the AgNPs and AuNPs LSPR absorption bands. The differential nature of this indicator makes it robust against external optical perturbations that affect the whole spectrum intensity, and it is useful for hydrogen peroxide detection purposes since the AuNPs LSPR band is stable enough to be used as an optical real-time reference. Figure 10 shows the evolution of the selected indicator (the difference between the normalized maximum absorbance of AgNPs LSPR and the AuNPs LSPR), showing a very similar behavior to the AgNP-only sensor previously presented. Figure 11 shows the calibration curve for 1, 10, 100, and 1000 ppm of H_2_O_2_. When the experimental results are plotted in a semi-logarithmic graph they show a good linear fitting, suggesting a logarithmic relationship between the optical fiber sensor’s response and the H_2_O_2_ concentration.

**Figure 12:**
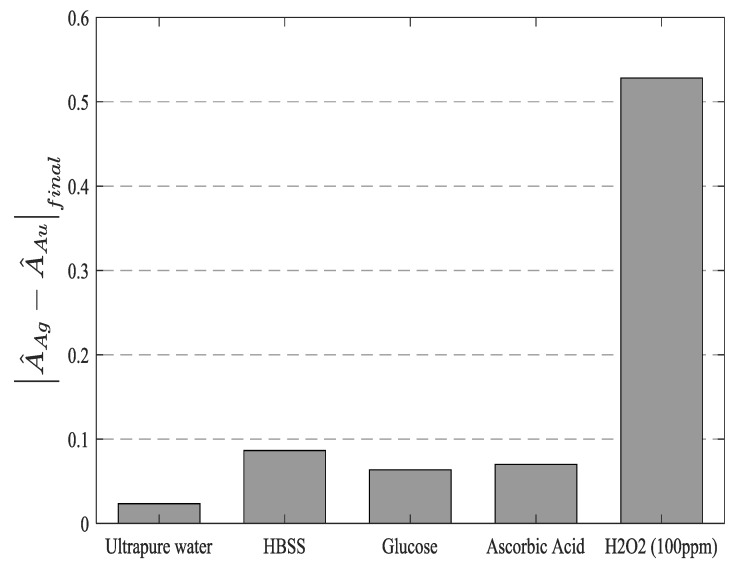
Cross-sensitivity of the sensors against common interferants. Hanks’ Balanced Salt Solution (HBSS), Glucose (100 mM) and Ascorbic Acid (400 ppm) were used in the cross-sensitivity test. It is shown that the response of the sensor can be considered specific to H2O2, since other reagents show readings that are more than 5 times lower, even when very high concentrations of the interfering compounds were used.

The response curves show a standard deviation as high as 0.0113 in normalized units, which gives an estimated LOD of 2.7 ppm, which is comparable to many works in the bibliography. The sensors showed a good selectivity towards the detection of hydrogen peroxide, can be seen in Figure 12. The experimental results reveal when the sensitive region is exposed to high concentrations of other interfering agents (glucose, ascorbic acid) or well to other solutions such as ultrapure water or HBSS, the response of the sensors was more than 5× lower than their response to 100 ppm of H_2_O_2_. One very important fact is that although the intensity of both peaks may vary significantly due to chemical interferants, temperature, or refractive index variations, the H_2_O_2_ sensing information is coded into the relative aspect of both peaks. Consequently, the sensor response remains stable as far as such interferences affect both LSPR peaks a similar amount, while giving significant readings when the sensors are exposed to Reactive Oxygen Species (ROS). This robustness relies on the ratiometric nature of this self-referenced approach. This promising result clearly indicates the high selectivity of this biosensing platform towards variable H_2_O_2_ concentrations, avoiding false positive measurements to other potential interfering compounds for H_2_O_2_ detection.

**Figure 13:**
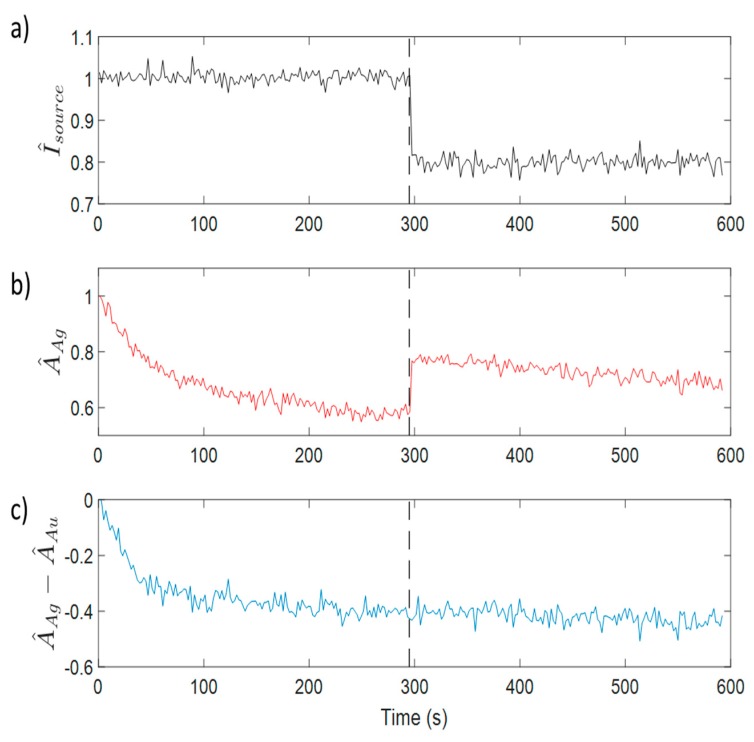
(**a**). Light intensity injected by the light source in the sensors. At 297 s into the experiment (dotted vertical line), an optical connector was loosened on purpose in order to decrease the light coming from the source by 30%. (**b**) Response of an AgLbL sensor. It is clearly shown how the source variation affects the signal, leading to a wrong peroxide reading. (**c**) Evolution of the differential indicator of a AgLbL+AuLbL sensitive coating. The signal remains almost intact in the case of the self-referenced sensor, even with such source variations.

Furthermore, the use of this final self-referenced indicator shows a very robust behavior compared to other approaches. As has been previously discussed, it is very common in this kind of intensity-based optical fiber sensor for the optical response to be studied by taking the optical reference at the beginning of the experiment. The differential nature of the proposed indicator helps to maintain the signal integrity even in difficult situations, such as when the experimental conditions are altered during the measurement. In Figure 13 a robustness test is shown to be carried out interrogating two sensors at the same time. During the experiment (time: 297 s), an optical connector has been loosened on purpose in order to induce an optical loss (around 30%) that simulates a dramatic change in the optical conditions of the experiment. In Figure 13a the variation of the light intensity coupled to the optical fiber sensors is shown. The graphs in Figure 13b show that the optical response of the AgNP-only LbL sensor suffers a significant variation, yielding a wrong peroxide concentration reading at the end of the experiment, since it is only based on the intensity of the absorbance signal. Figure 13c shows the differential measurement available in the mixed AgNP AuNPs LbL coatings. It is clearly visible that the information contained in such indicator remains consistent even during the occurrence of such dramatic event, and the peroxide reading at the end of the experiment is almost unaffected by the modification of the experimental conditions. 

## 4. Conclusions

In this work, a simple and robust optical fiber sensor is presented for the detection of small concentrations of hydrogen peroxide, which is a pathological analyte present in a wide variety of damage mechanisms. The Layer-by-Layer nanoassembly has been used as a tool for the fabrication of the sensitive nanocoatings onto the optical fiber core because this nanofabrication technique makes immobilization of two different types of metallic nanoparticles such as AgNPs and AuNPs into the multilayer LbL films possible. The presence of both types of nanoparticles is of great interest for biosensing applications because they show interesting optical properties inherent to their LSPR absorption bands, thereby showing a remarkable difference in sensitivity to the analyte of study. According to this outcome, after exposure to different H_2_O_2_ concentrations, the LSPR band associated to AuNPs remained stable nearly at the same maximum absorbance for all H_2_O_2_ concentrations, whereas the maximum absorbance of the AgNPs was decreased by a high magnitude when the analyte concentration was increased. In this work a differential estimation of the responses of the optical fiber sensors is proposed that provides a stable real-time reference that can be extracted directly from the spectral response of the optical fiber sensor. This differential self-reference measurement shows an extraordinary robustness, even to dramatic experimental changes during the measurements, which make them virtually immune to light source fluctuations, or optical fiber setup movements. The estimated limit of detection (LOD) is 2.7 ppm of hydrogen peroxide.
